# The possible impact of novel mutations in human papillomavirus 52 on the infection characteristics

**DOI:** 10.1099/mgen.0.000962

**Published:** 2023-04-27

**Authors:** Yingxin Gong, Yan Wang, Qi Zhou, Wenjie Qu, Fang Chen, Yaping Wang, Jiayin Mo, Hongwei Zhang, Lin Lin, Tianyi Bi, Xujie Wang, Jiashi Gu, Long Sui, Yanyun Li

**Affiliations:** ^1^​ Department of Gynecology and Obstetrics, Obstetrics and Gynecology Hospital of Fudan University, Shanghai, PR China; ^2^​ Department of Obstetrics and Gynecology, Shanghai Changning Maternity and Infant Health Hospital, Shanghai, PR China; ^3^​ Department of Obstetrics and Gynecology, Shanghai Pudong Hospital of Fudan University, Shanghai, PR China

**Keywords:** Human papillomavirus, variation, persistent infection, multiple infection, vaccine breakthrough infection, cervical cancer

## Abstract

Human papillomavirus 52 (HPV52) infection is prevalent in the Chinese population, and variations in HPV52 show correlations with oncogenicity. However, no specific variation in HPV52 was reported to show relevancy to infection characteristics. In this study, we retrieved 222 isolates of E6 and L1 full-length genes from 197 Chinese women with HPV52 infection. After sequence alignment and phylogenetic tree construction, we found that 98.39 % of the collected variants belonged to the sublineage B2 and two variants displayed incongruence between the phylogenetic tree of E6 and L1. The analysis of the infection pattern showed that the presence of C6480A/T mutation in the L1 gene was associated with single infection (*P*=0.01) and persistent infection (*P*=0.047) of HPV52, while the A6516G nucleotide change was relevant to transient infection (*P*=0.018). Our data also indicated that variations T309C in the E6 gene and C6480T, C6600A in L1 were more commonly presented in patients with high-grade cytology (*P*<0.05). One HPV52 breakthrough infection after vaccination was identified, which hinted at the immune escape post-vaccination. Young coitarche age and non-condom usage were correlated to multiple infections. This study provided insight into the polymorphism of HPV52 and revealed the impact of variations in HPV52 on its infection characteristics.

## Data Summary

**Fig. 1.**
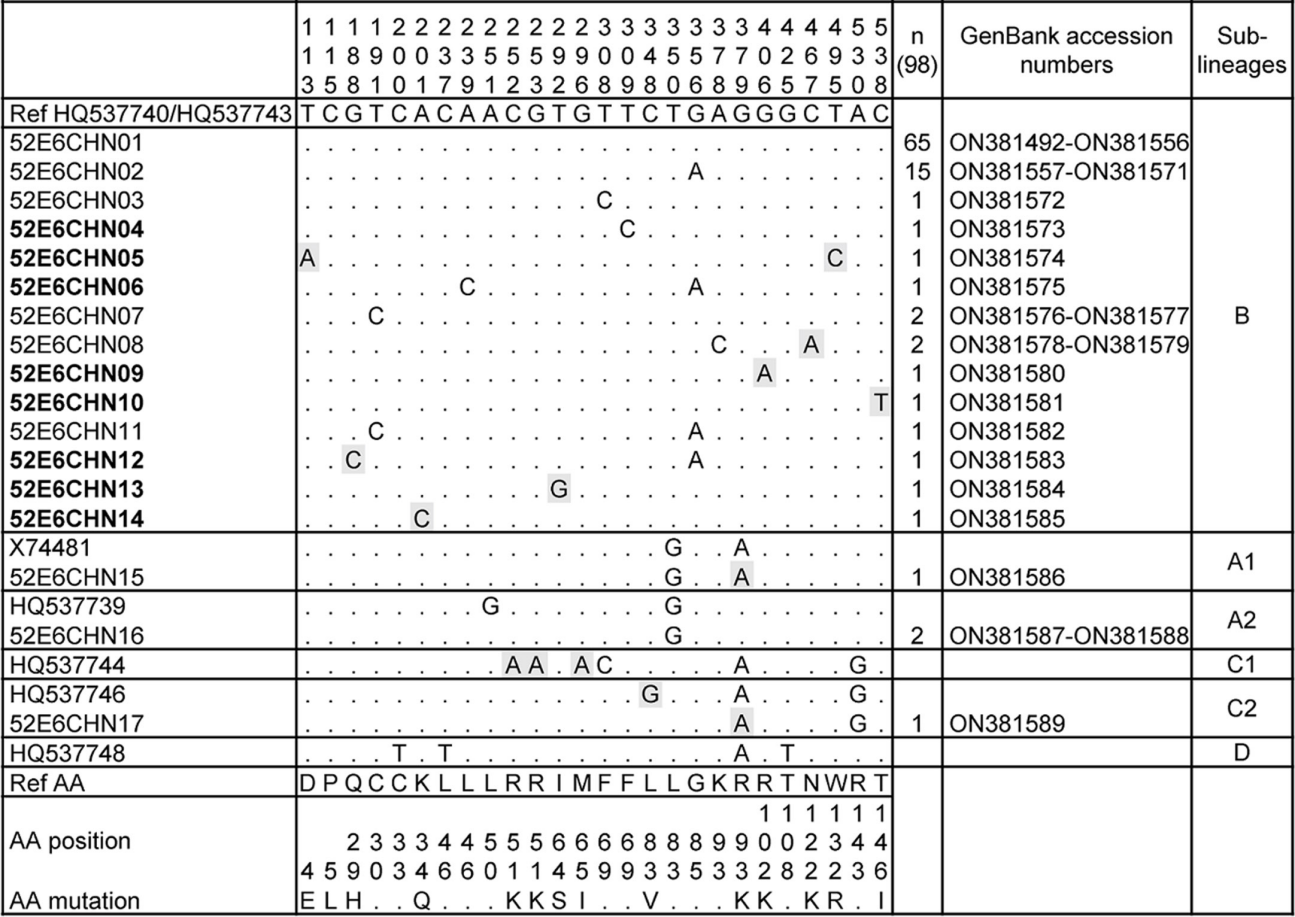
Genetic variability of HPV52 E6 nucleotide sequences. Note: numbering refers to the sequence of the HPV52 lineage B prototype reference sequence (GenBank: HQ537740/HQ537743). Each row indicates the isolate identification and the nucleotide sequence alignment compared to the reference. Novel HPV52 variants are highlighted in bold and novel nucleotide substitutions are highlighted in grey.

**Fig. 2.**
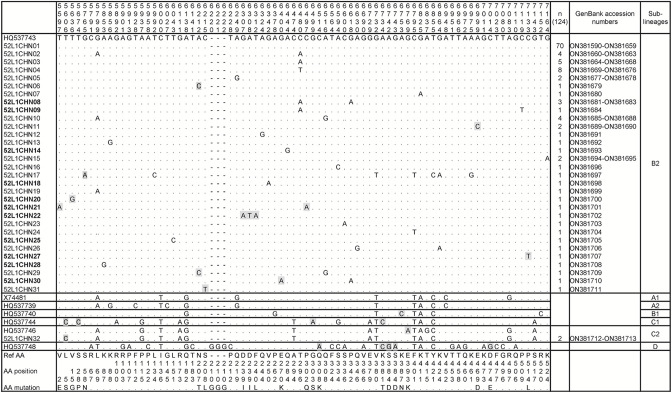
Genetic variability of HPV52 L1 nucleotide sequences. Note: numbering refers to the sequence of the HPV52 lineage B2 prototype reference sequence (GenBank: HQ537743). Each row indicates the isolate identification and the nucleotide sequence alignment compared to the reference. Novel HPV52 variants are highlighted in bold and novel nucleotide substitutions are highlighted in grey.

GenBank accession numbers of the samples are available in Figs 1 and 2, and the sequences can also be obtained in Table S1 (available with the online version of this article). The reference sequences used are available from GenBank, and the accession numbers are available in Table S1.

**Impact Statement**

Human papillomavirus (HPV) is a pathogen that relates to carcinogenesis in the cervix, anus, and oropharynx with high mortality. Nowadays, various research on phylogeny and variation of HPV has revealed its association with pathogenicity and infectivity. However, there are insufficient data for HPV52 compared to HPV16/18, and prevailing variants associated with infection characteristics are rarely noticed. We expanded the sequence data of HPV52 from the Chinese mainland and clarified the variants that might be in relation to cervical lesion severity and infection characteristics. Our study provided basic data for developing therapeutic protocols and vaccines for HPV infection in China. It also provided a theoretical basis and support for HPV recombination.

## Introduction

Human papillomavirus (HPV) arouses extensive attention for its oncogenic potential, and 15 high-risk genotypes with a correlation with cervical cancer have been identified. The distribution of HPV genotypes varies among regions, typically represented by the significantly high prevalence of HPV52 and HPV58 in Asia [1]. According to the latest report published by the Catalan Institute of Oncology (ICO) and the International Agency for Research on Cancer (IARC), the prevalence of HPV52 in women without lesions and women with precancerous cervical lesions including low-grade squamous intraepithelial lesion (LSIL) and high-grade squamous epithelial lesion (HSIL) ranked only second to HPV16 in the world, especially in less developed regions [2]. In China, the prevalence of HPV52 in cervical LSIL even exceeded HPV16 and the overall genotype distribution in the total population showed the indistinguishable prevalence of HPV52 and HPV16 in western China [3, 4]. Although the study on HPV16/18 remains highlighted, more and more epidemiological evidence is pushing HPV52 to the centre stage of HPV research.

Diverse intratype HPV variants have been identified with sequences included in the GenBank database. Based on tree topology and nucleotide sequence differences, the variants of the same genotype are further classified as lineages (1–10 %) and sublineages (0.5–1 %). Four lineages have been identified in HPV52 and lineages A, B, and C each branch into two sublineages. The variation between lineages leads to the disparity in infection and cancerous capacity. The L1 capsid protein plays a major role in mediating the entry of the virus, thus initiating the infection. While E6 and E7 proteins modulate the oncogenic pathway by interfering with p53 and Rb, contributing to tumorigenesis. The Asia-prevailing lineage B of HPV52 exhibited a higher potential to induce severe cervical lesions compared to lineage A [5]. In addition, evidence of specific mutations including K93R (A379G) in HPV52 E6 and C632T (T20I) and G760A (G63S) in HPV58 E7 that contributed to precursor lesions of cancer have been revealed [6, 7]. The genetic variability of HPV casts a great challenge on disease prevention and treatment.

Most HPV infection results in spontaneous clearance, whereas 10–20 % of infection persist latently [8]. At present, there is no generally recognized definition for persistent HPV infection. The most common definition is ≥2 consecutive positive HPV DNA tests (regardless of HPV type) and the minimum duration of HPV persistence is 6–12 months [9, 10]. Persistent infection is closely correlated to the incidence and progression of cervical precancerous lesions and cancer. HPV E6 and E7 oncoproteins were identified to interfere with sensors, adaptors, and signalling molecules, thus altering innate immune pathways, and contributing to persistent infection [11]. The role of HPV genomic polymorphisms in characterizing infection is well-interpreted by the persistent tendency of non-European HPV16 variants [12, 13]. The prevalence of variation T350G (L83V) in HPV16 E6 in the population of persistent infection was found, albeit controversial, as the conflicting result was also retrieved [14–17]. Similarly, HPV52 lineage C variants displayed a slower clearance than lineage A [18], and non-prototype long control region (LCR) was found to be correlated with HPV52 persistence [19]. However, no specific variation in HPV52 was reported to shape the infection pattern. Cervical cytology and HPV-testing are the foundation of cervical cancer screening procedures, and the cytology alteration serves as the first step of HPV-related malignancy transformation. However, whether polymorphisms in the HPV52 genome contributed to the cytological progression varied among reports [7, 20, 21]. HPV prophylactic vaccines provided protection for susceptible populations, whereas the occurrence of breakthrough infections was noteworthy [22, 23]. The HPV persistence and vaccine escape are main concerns for cervical cancer prevention, and more clinical data are requisite for further research.

Considering the high prevalence of HPV52 in China and the lack of data and research on variants related to infection characteristics, we analysed the nucleotide sequences of prevailing HPV52 variants to investigate the relevancy between variations and HPV infection status, as well as the severity of the cervical lesions. In addition, the clinical features of women with HPV persistent infection and vaccine breakthrough infection were also analysed to provide information for future investigation.

## Methods

### Study population and sample collection

From March 2021 to March 2022, a total of 197 HPV52-positive cervical samples were collected from the colposcopy clinic at the Gynaecology and Obstetrics Hospital of Fudan University. The study was approved by the Ethical Committee of Gynaecology and Obstetrics Hospital of Fudan University and informed consent was taken from each patient. Those patients were admitted to the colposcopy considering abnormal results of liquid-based cytology tests (LCT), HPV testing, or cervical treatment history. The cervical exfoliated cells were collected with the sampling brush and stored in cell preservation solution. The coloscopy was performed by colposcopists who had over 5 years of experience in colposcopic diagnosis. Punch biopsies under colposcopy were performed in suspicious areas based on acetowhite and Schiller tests. The histopathological diagnoses were made by two senior pathologists. The clinical features including HPV genotype, HPV infection duration, LCT result, cervical histopathological result, age, medical history, geography, Ob/Gyn history, HPV vaccination history, age of coitarche, number of sex partners and contraception were recorded. The cytologic results were classified as negative for intraepithelial lesion or malignancy (NILM), atypical squamous cell of undetermined significance (ASC-US), low-grade squamous intraepithelial lesion (LSIL), atypical squamous cell cannot exclude HSIL (ASC-H), and high-grade squamous intraepithelial lesion (HSIL).

### DNA extraction, PCR amplification and sequencing

Genomic DNA was extracted from the cervical exfoliated cells using TIANamp Genomic DNA Kit (TIANGEN) and collected in 50 µl nuclease-free water, then stored at −80 °C. To amplify the full-length gene sequences of HPV52 E6 and L1, the type-specific primers were designed. The sequences of primers were as follows: HPV52 E6 gene, sense 5′-AGACCGAAACCGGTGTATATATATAGA-3′, anti-sense 5′-CCACACCATCTGTATCCTCCTCA-3′, and HPV52 L1 gene, sense 5′-TCCATTGAGTCAGGTCCTGACAT-3′, anti-sense 5′-ACATGCAAACAACACAGTACACACA-3′. PCR reactions were done in a 50 µl reaction volume containing one unit of TaKaRa Ex Taq (Takara, Japan), 1×Ex Taq Buffer (Mg^2+^ plus), 200 µM of dNTP Mixture and 20 pmol of each primer. The thermal cycling parameters were 98 °C for 10 s, 55 °C for 30 s and 72 °C for 40 s (E6)/90 s (L1), with a final extension in 72 °C for 7 min. PCR amplicons were separated on 1.2 % agarose gels and visualized by YeaRed Nucleic Acid Gel Stain staining under UV transillumination. A positive control of HPV52 plasmid and a negative control without template DNA were performed in each set of PCR. After purification, DNA amplicons were sequenced by ABI 3730xl DNA Analyzer using the same PCR primers. All samples had their PCR repeated in duplicate and were sequenced from both directions to exclude PCR artefacts, and the consensus sequences were obtained with Phred Quality Score higher than 20.

### Phylogenetic analysis and variant identification

The phylogenetic analysis was conducted in mega v11.0 following the guideline for phylogenetic reconstruction [24]. The HPV52 standard sequences of each lineage and sublineage (A1: X74481, A2: HQ537739, B1: HQ537740, B2: HQ537743, C1: HQ537744, C2: HQ537746, D: HQ537748) were all downloaded from GenBank database, NCBI. The reference genomes for HPV52 lineages and sublineages were referred to the publication of Burk *et al*. [25]. All obtained sequences (including 98 HPV52 E6 sequences and 124 L1 sequences) were aligned with the HPV52 prototypes by ClustalW and muscle, and pairwise distance was calculated in the Bootstrap method to consolidate the items with the same sequence and estimate p-distance. Phylogenetic trees were constructed based on the nucleotide sequences using the maximum likelihood method in mega 11.0 with 1000 bootstrap replicates, and the pairwise comparison was visualized in Microsoft Excel.

### Statistical analysis

The Independent-samples t-test was applied to compare the difference in the continuous variables between two groups and Levene’s test was applied to conduct a homogeneity test of variance. Mann-Whitney Rank sum test was implemented to evaluate the difference in histopathology between the two groups. Pearson χ^2^ and Fisher’s exact test were employed to evaluate the distribution of HPV52 mutations to HPV infection status and disease severity. *P*<0.05 was considered statistically significant. Statistical analysis was conducted in SPSS v25.0 and visualized by Microsoft Excel.

## Results

### Demographic characteristics of the population and risk association with HPV infection patterns

A total of 197 cervical swabs from women with HPV52 infection were collected and complete clinical records were obtained from 130 patients. The median age of these subjects was 45 years (range 23–74). The Ob/Gyn history showed that 37.69 % (49/130) of women were post-menopausal, and 17.69 % (23/130) of women were nullipara. Among LCT results of all subjects, 57.69 % (75/130) were cytologic NILM, 19.23 % (25/130) were cytologic ASCUS, 16.15 % (21/130) were LSIL, 6.92 % (9/130) were high-grade cytology (ASC-H +HSIL). Regarding biopsy histopathology, 13.08 % (17/130) of patients were HSIL or cancer, with 39.23 % (51/130) identified as normal. In terms of HPV infection pattern, 36.15 % (47/130) of women experienced multiple infections, and 61.54 % (80/130) of infections were persistent (> 1 year). The alternation of HPV genotype was observed in 19 women. As shown in Table S2, 130 subjects were each divided into three group pairs based on the pattern of HPV infections to clarify the association with demographic characteristics. Younger coitarche age (*P*=0.021) and a lower proportion of condom usage (*P*=0.045) were observed in the multiple infection population. Older average age (*P*=0.023) in the persistent infection population and a higher proportion of multiple infections (*P*=0.002) in the genotype alteration group were also impressive.

A total of ten women were vaccinated with the HPV prophylactic vaccine. Five women received the nonavalent vaccine, four received the quadrivalent vaccine, and one received the bivalent vaccine. All the vaccinated women received their vaccination after their coitarche. Compounding the situation, five women were HPV-positive pre-vaccination, and three women did not receive HPV testing before vaccination so their pre-vaccination conditions were unclear. Two patients were negative for HPV before vaccination, one of which received the quadrivalent vaccine and was infected with HPV52 9 months after vaccination completion. The other patient had a 2 month history of HPV39, HPV52 co-infection with cytological NILM, and finished three-dose nonavalent vaccination at the age of 26. Interestingly, one woman had HPV58 infection before the vaccination, which turned negative and subsequently became HPV52 positive after the inoculation.

### Variations of E6 and L1 genes

From the 130 patients with complete clinical records, 98 E6 and 124 L1 full-length sequences were retrieved. The gap between samples and isolates may be explained by the low viral titre or unstable amplicons. We obtained 32 distinct L1 variants and 17 E6 variants of HPV52, which were all submitted to the GenBank database and received the accession numbers. Compared to the sublineage B2 prototype reference sequence (GenBank: HQ537743), 33.67 % (33/98) of the E6 isolates and 43.55 % (54/124) of the L1 isolates showed nucleotide mutations. And eight novel E6 variants and 11 novel L1 variants were also identified. A summary of nucleotide and amino acid sequence variation throughout the E6 and L1 fragments were shown in Figs 1 and 2, respectively.

In the E6 gene, 17 nucleotide substitutions were identified with nine novel variations and nine non-synonymous substitutions resulting in amino acid change. Two non-synonymous substitutions appeared more than once, including G379A (R93K) and C467A (N122K). The synonymous nucleotide substitution G356A appeared in 17 isolates and was specified in the B lineage. In the L1 gene, 45 nucleotide substitutions were identified with 13 novel variations and 13 non-synonymous substitutions, in which T5606C (L5S), A6212C (N207T), and A6999C (E469D) appeared in more than one isolate. Synonymous substitutions C6480A/T were observed in 21 L1 isolates, among which 13 were C6480A and eight were C6480T. In addition, the substitution in nucleotide 6480 was only seen in the B2 sublineage. Another synonymous substitution G5799A appeared in 11 isolates, which also occurred in prototype sequences of lineage A and sublineage C2. Four isolates had more than two substitutions after being aligned with the reference sequence.

### Variation frequency of E6 and L1 genes

In this study, the mutation frequency was 33.67 % (33/98) for E6 and 43.55 % (54/124) for L1. The mutation rate was 0.41 (40/98) per isolate for E6 and 0.83 (103/124) per isolate for L1 in HPV52. The proportion of polymorphic nucleotides was greater in the E6 than in the L1 gene, as 17 variation sites were identified over 447nt (3.80 %) in E6 and 45 variation sites over 1590nt (2.83 %) in L1.

### Phylogenetic tree

**Fig. 3.**
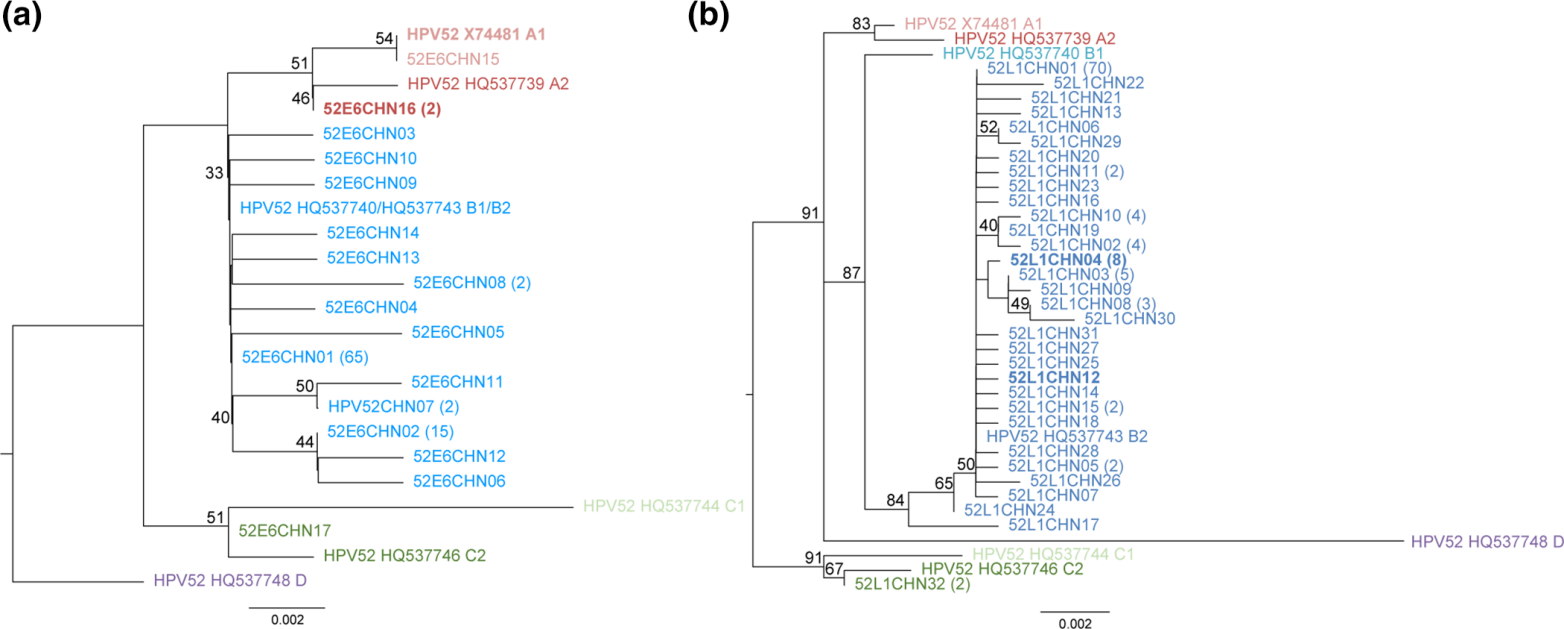
Phylogenetic tree of the HPV52 variants. Maximum likelihood analysis (with mega 11.0 programme) of E6 (**a**) and L1 (**b**) nucleotide sequences were inferred from obtained HPV52 variants and seven reference sequences. The numbers below branches indicate bootstrap values. Lineages A, B, C, and D were marked in red, blue, green and purple, respectively. The bold variants were sequenced from two samples that shared the same E6 sequence and showed phylogenetic incongruence between E6 and L1 trees.

As shown in Fig. 3, maximum likelihood phylogenetic trees and pairwise comparison based on the L1 and E6 were inferred from obtained HPV52 variants and seven reference sequences. Lineages A, B, C, and D were marked in red, blue, green and purple, respectively. In total, 98.39 % (122/124) of variants belonged to B2 sublineages with 56.45 % (70/124) of L1 isolates sharing the same sequence with the B2 prototype. The remaining two variants belonged to the C2 sublineage. No isolates clustered in the branches of A and D lineage. And no novel lineage or sublineage was found based on the phylogenetic tree and pairwise distance comparison. In the 98 E6 isolates obtained, 66.33 % (65/98) of isolates shared the same sequence with the lineage B prototype as the E6 gene of sublineage B1 and B2 were identical. Consistent with the L1 gene, 96.94 % (95/98) of E6 isolates belonged to lineage B. However, variants from two samples displayed incongruence between the phylogenetic trees of E6 and L1 as illustrated in bold. They belonged to the B2 sublineage in the phylogenetic tree based on the L1 sequence, while they belonged to the A2 sublineage in the E6 tree.

### Distribution of E6 and L1 variation in different HPV infection patterns and cervical lesions

As seen in Table 1, the presence of T309C in E6 variants exhibited a significant difference in the distribution of cytology compared to other variations (*P*=0.043), but no correlation was shown (OR=1.333, 95 % CI: 0.757–2.348). There were no differences in cytology distribution in other E6 variations (*P*>0.05). The variations in E6 showed no correlation with multiple, persistent HPV infections and the histopathological result of cervical biopsy (*P*>0.05).

**Table 1.** Distributions of HPV52 E6 variations in HPV infection pattern and cervical histopathology

Nucleotide	Amnio acid	Single	Multiple	*P*	Transient	Persistent (>1 year)	*P*	NILM	Ascus	LSIL	HSIL	P*
T113A	D4E	0	1	0.357	1	0	0.388	1	0	0	0	1
G188C	Q29H	0	1	0.357	0	1	1	0	1	0	0	1
T191C	C30C	1	2	0.600	0	3	0.425	1	1	0	1	0.124
A201C	K34Q	0	1	0.357	1	0	0.388	0	0	1	0	1
A239C	L46L	1	0	1	1	0	0.388	0	0	1	0	1
T292G	I64S	0	1	0.357	0	1	1	1	0	0	0	1
T308C	F69F	1	0	1	0	1	1	0	1	0	0	1
T309C	F69F	1	0	1	1	0	0.388	0	0	0	1	**0.043**
T350G	L83L	2	1	1	1	2	1	2	0	0	1	0.124
G356A	G85G	11	7	0.756	9	9	0.279	10	3	4	1	1
A378C	R93K	1	1	1	0	2	0.52	1	1	0	0	0.124
G379A	R93K	2	0	0.536	0	2	0.52	2	0	0	0	1
G406A	R102K	1	0	1	1	0	0.388	1	0	0	0	1
C467A	N122K	1	1	1	0	2	0.52	1	1	0	0	1
T495C	W132R	0	1	0.357	1	0	0.388	1	0	0	0	1
A530G	R143R	1	0	1	0	1	1	1	0	0	0	1
C538T	T146I	1	0	1	0	1	1	1	0	0	0	1
Total		63	35		38	60		53	19	18	4	

* HSIL vs NILM+ASCUS+LSIL. *P* values were calculated by Pearson χ2 and Fisher’s exact test based on the expected value.

ASC-H, atypical squamous cells, cannot exclude HSIL; ASC-US, atypical squamous cells of undetermined significance; HPV, human papillomavirus; HSIL, high-grade squamous intraepithelial lesion; LSIL, low-grade squamous intraepithelial lesion; NILM, negative for intraepithelial lesion or malignancy.

Table 2 summarized the variations that occurred more than twice in L1 and the relationship to infection pattern and cervical pathology. The variations in the L1 gene showed a significant correlation with HPV infection patterns. The presence of C6480A in L1 showed a significantly negative correlation with multiple infections (*P*=0.01. OR=0.835, 95 % CI: 0.758–0.921), represented by the status of single infection in all 13 samples with the variation. In addition, a total of 21 isolates with substitutions in the nucleotide 6480 were all single infections and exhibited a more significant correlation (*P*<0.001). However, another substitution C6480T showed no significant correlation with multiple infections (*P*=0.068), but it displayed positive correlations with persistent infection no matter longer than 1 year (*P*=0.047, OR=1.119, 95 % CI:1.035–1.210) or 2 years (*P*=0.019, OR=1.171, 95 % CI:1.032–1.330). On the contrary, the presence of A6516G exhibited a significantly negative correlation with persistent infection (*P*=0.018, OR=0.898, 95 % CI:0.817–0.987). The substitution C6480T also exhibited a significantly positive correlation with high-grade cytology (*P*=0.035, OR=12.111, 95 % CI:1.691–86.752). Similarly, the variation C6600A in L1 showed a significant difference in the distribution of cytology compared to other variants (*P*=0.042), but no correlation was shown (OR=1.250, 95 % CI: 0.806–1.938). The variations in L1 showed no correlation with the severity of the histopathological results (*P*>0.05).

**Table 2.** Distributions of HPV52 L1 variations in HPV infection pattern and cervical histopathology

Nucleotide	Amnio acid	Single	Multiple	*P*	Transient	Persistent (>1 year)	*P*	NILM	Ascus	LSIL	HSIL	P*
G5799A	L69L	8	3	0.747	4	7	1	8	0	3	0	1
C6480A	T296T	13	0	**0.01**	3	10	0.2	10	1	2	0	1
C6480T	T296T	8	0	0.07	0	8	**0.047**	3	2	1	2	**0.035**
A6516G	Q308Q	4	1	0.765	5	0	**0.018**	3	0	2	0	1
G6618A	P342P	3	1	1	1	3	0.933	3	0	1	0	1
G6732T	V380V	1	2	0.617	0	3	0.413	3	0	0	0	1
C6795T	F401F	1	3	0.268	0	4	0.261	3	0	1	0	1
G6825A	K411K	2	1	1	0	3	0.413	2	1	0	0	1
T6855C	Y421Y	1	2	0.617	0	3	0.413	3	0	0	0	1
**Total**		79	45		49	75		70	25	20	5	

* HSIL vs NILM+ASCUS+LSIL. *P* values were calculated by Pearson χ2 and Fisher’s exact test based on the expected value.

ASC-H, atypical squamous cells, cannot exclude HSIL; ASC-US, atypical squamous cells of undetermined significance; HPV, human papillomavirus; HSIL, high-grade squamous intraepithelial lesion; LSIL, low-grade squamous intraepithelial lesion; NILM, negative for intraepithelial lesion or malignancy.

## Discussion

The gene sequence of HPV is the basis of the infectivity and pathogenicity of HPV. This study investigated the correlation between HPV52 variations and HPV infection characteristics, in addition to lesion severity. We identified 17 variations in the E6 gene and 45 variations in the L1 gene. The presence of C6480A/T in the L1 gene was found to be associated with single and persistent infection, while the A6516G was relevant to transient infection. The presence of C6480T in L1 showed positive correlations with the high-grade cytology. In terms of infection characteristics, we found that young coitarche age and non-condom usage were correlated to multiple infection and multiple infection seemed to be correlated to genotype alteration.

We retrieved 124 L1 sequences and 98 E6 sequences from 130 cervical samples. The phylogenetic tree based on the L1 gene was the generally acknowledged genotyping method. Among the obtained isolates, 98.39 % of variants belonged to sublineage B2 and 1.61 % of variants belonged to the sublineage C2 based on the L1 tree. The prevalence of HPV52 sublineage B2 was confirmed by previous publications, represented by the large-scale study involving specimens from 14 sites worldwide revealing its great dominance in Asia, which reached 89.0 % [5]. The prevalence of sublineage B2 was further verified by two studies performed in two Asian countries, Korea (91 isolates) [7] and southwest China (53 isolates) [26]. Our study analysed the lineage attribution of 124 isolates with full-length L1 gene and collaboratively evidenced the prevalence of lineage B2 in China. The incongruence of E6 and L1 genes in the phylogenic tree was observed in two variants. One isolate was retrieved from a single infection with HPV52, the other was from the multiple infection with HPV52 and HPV39. The phylogenetic incongruence between early and late genes of alpha-HPV have been identified decades ago, which was most pronounced by E6 and L2 [27, 28]. The incongruence revealed different evolutionary pathways, which might result from the breakage of the gene, intensive selection, and recombination [29]. The practical circumstance of the variants in our study cannot be affirmed as we didn’t obtain the whole genome, but it shall offer enlightenment to the possibility of HPV recombination [30].

In this study, the proportion of polymorphic nucleotides coincided with previous reports of Alpha-9 species. The proportion of variable nucleotide sites was estimated as 4.4 % across the 7993nt HPV52 genome by Chen *et al*. [31], which ranged from 2.5–4.5 % for E6 and 2.20–3.8 % for L1 from various reports [6, 7, 26, 32]. The percentages of variable nucleotide positions across HPV16 and HPV18 showed similar results [33, 34]. We also identified nine novel variations in E6 and 13 in L1, which have not been reported previously. These latest-found variations included T113A (D4E), G188C (Q29H), A201S (K34Q), A239C, T292G (I64S), T309C, G406A (R102K), T495C (W132R), C538T (T146I) in E6; T5597A (V2E), T5636G (V15G), A5853G, T6043C, G6307A (D239I), A6308T (D239I), T6312Z (F240L), G6342A, G6400A (E270K), A6444G, C6480A/T, C6497A (P302Q), C1541T (P514L) in L1. We did not detect the secondary structure alteration of the protein correlated with nonsynonymous nucleotide substitutions in E6 and L1. Most substitutions in the L1 gene were synonymous. The four variations in L1 with the most frequent occurrences were C6480A, G5799A, C6490T, and K6516G. The nucleotide synonymous substitution G356A in E6 was the most frequent variation compared to prototype B and specifically appeared in lineage B. Although synonymous substitution did not alter the protein sequence, it had been shown that synonymous codon mutations affected transcription modifications, translation, RNA folding and splicing, thus impacting cellular processes [35]. In addition, the synonymous substitution T309C in E6 and C6600A in the L1 exhibited distributions in high-grade cytology. Since the two variations both occurred only once, their correlation with the severity of cytological results was not sufficient. However, the substitution C6480T in L1 exhibited a significantly positive correlation with high-grade cytology, which hasn’t been identified previously. Synonymous changes in L1 could have impacted a viral regulatory element since the L1 sequence is not exclusively a coding sequence. Those synonymous mutations may alter the pathogenicity by modulating the transcription and RNA modification, which merits further study.

The association analysis of clinical features showed that early coitarche and non-condom usage might be vulnerable factors to multiple infections, while multiple infections were not related to persistent infection and the severity of the cervical lesion. The finding contrasted with some perspectives considering that multiple HPV infections were associated with infection persistence and the incidence of the precancerous lesion [36, 37]. However some publications demonstrated that the multiple infections of non-16/18 hrHPVs did not increase the incidence of HSIL and cancer [38]. The number of subjects was small in our study with only 15 women with HSIL and two with cancer, resulting in the inadequacy of correlation analysis for lesion severity. During the analysis of variations, we accidentally found that the synonymous substitutions in E6 excluding A530G all occurred in samples with HPV52 single infection. But no variation in E6 showed a correlation with HPV infection status. Nevertheless, the presence of C6480A in L1 variants was significantly correlated with HPV52 single infection, and L1 variants with substitution in nucleotide 6480 were all from samples with single infections. Most previous studies merely focused on variants from single HPV infection, and little research studied the relationship between variations and multiple infections. The mechanism behind multiple infections is still controversial, the possibility of synergistic and competitive interactions between different genotypes has been put forward [39, 40]. Our study revealed the potential of the autogenous regulation of HPV infection, which might contribute to its competitive advantages over other genotypes. HPV persistence contributes to the progress of cervical lesions and serves as the risk factor for cervical cancer. The relevancy between persistent infection and older age was presented, which could be attributed to the decline in immune function. The link between variation and HPV persistence was reported by Aho *et al*., and a nonprototypic LCR variant was identified as the only independent predictor of HPV52 persistence, resulting in the loss of a binding site for a repressor of HPV expression [19]. Although a previous study suggested the irrelevance of L1 polymorphism to HPV52 persistence [32], our findings provided the possibility that variations in the L1 gene impacted HPV persistence. The variation C6480T served as a promoter for persistent infection, while A6516G acted as a protector, which prevented HPV persistence. The variations in HPV52 E6 showed no correlation with HPV persistence, which coincided with the previous publication [19].

Vaccine escape has become a hot topic as the infection of SARS-CoV-2 after vaccination was common because of variation [41, 42]. Ten vaccinated patients with HPV52 infection were found. All women received their first dose after coitarche, which had been proven to be associated with the infection of vaccine types [22]. The patient who experienced the HPV58 infection before vaccination and was replaced by HPV52 after vaccination infection displayed an intriguing example of genotype alteration. We considered those who were negative for HPV before vaccination and got infected with vaccine-covered HPV genotypes after receiving vaccines as vaccine escape populations. Only one case can be identified as HPV52 breakthrough infection in this study, and the woman developed multiple infections of nonvaccine type HPV39 in addition to HPV52. Although the reduction in the neutralizing litres was possible, her smoking habit might also confer a high risk for breakthrough infection.

The research on variations in HPV16/18 has been widely studied and numerous sequences have been submitted to the GenBank database. By contrast, the number of HPV52 sequences in the collection was much fewer although its prevalence in Asia has been well-recognized. In this study, we tripled the number of E6 and L1 sequences of HPV52 from China mainland in the GenBank database and provided valuable information on the genomic diversity of HPV52. However, there were also some limitations. Firstly, most patients were from Shanghai and Jiangsu province, which made it difficult to analyse the geographic correlation. Secondly, most variations only occurred once, whose clinical significance cannot be well-interpreted. Further research will be required for investigating the concrete functions of those synonymous substitutions.

## Conclusion

In the light of our findings, the synonymous C6480A/T variant in HPV52 L1 is correlated with single and persistent HPV52 infection, while A6516G is more frequently found in transient HPV52 infection. Also, young coitarche age and non-condom usage are correlated to multiple infection. The study provides insights for future studies on epidemiology, phylogenetics, pathogenicity, HPV infection pattern, and HPV vaccine escape.

## Supplementary Data

Supplementary material 1

Supplementary material 2

## References

[1] Bruni L, Diaz M, Castellsagué X, Ferrer E, Bosch FX (2010). Cervical human papillomavirus prevalence in 5 continents: meta-analysis of 1 million women with normal cytological findings. J Infect Dis.

[2] Bruni L AG, Serrano B, Mena M, Collado JJ, Gómez D (2022). Human papillomavirus and related diseases in the world.

[3] Zhang J, Cheng K, Wang Z (2020). Prevalence and distribution of human papillomavirus genotypes in cervical intraepithelial neoplasia in China: a meta-analysis. Arch Gynecol Obstet.

[4] Chen L, Dong Y, Li J, Zhao J, Wang D (2021). The genomic distribution map of human papillomavirus in Western China. Epidemiol Infect.

[5] Zhang C, Park J-S, Grce M, Hibbitts S, Palefsky JM (2014). Geographical distribution and risk association of human papillomavirus genotype 52-variant lineages. J Infect Dis.

[6] Ding T, Wang X, Ye F, Cheng X, Ma D (2010). Distribution of human papillomavirus 58 and 52 E6/E7 variants in cervical neoplasia in Chinese women. Gynecol Oncol.

[7] Choi YJ, Ki EY, Zhang C, Ho WCS, Lee S-J (2016). Analysis of sequence variation and risk association of human papillomavirus 52 variants circulating in Korea. PLoS One.

[8] Shanmugasundaram S, You J (2017). Targeting persistent human papillomavirus infection. Viruses.

[9] Rositch AF, Koshiol J, Hudgens MG, Razzaghi H, Backes DM (2013). Patterns of persistent genital human papillomavirus infection among women worldwide: a literature review and meta-analysis. Int J Cancer.

[10] Hoffman SR, Le T, Lockhart A, Sanusi A, Dal Santo L (2017). Patterns of persistent HPV infection after treatment for cervical intraepithelial neoplasia (CIN): a systematic review. Int J Cancer.

[11] Gusho E, Laimins L (2021). Human papillomaviruses target the DNA damage repair and innate immune response pathways to allow for persistent infection. Viruses.

[12] Sanchez GI, Kleter B, Gheit T, van Doorn L-J, de Koning MNC (2011). Clinical evaluation of polymerase chain reaction reverse hybridization assay for detection and identification of human papillomavirus type 16 variants. J Clin Virol.

[13] Tornesello ML, Losito S, Benincasa G, Fulciniti F, Botti G (2011). Human papillomavirus (HPV) genotypes and HPV16 variants and risk of adenocarcinoma and squamous cell carcinoma of the cervix. Gynecol Oncol.

[14] Lee K, Magalhaes I, Clavel C, Briolat J, Birembaut P (2008). Human papillomavirus 16 E6, L1, L2 and E2 gene variants in cervical lesion progression. Virus Res.

[15] Zhang L, Yang B, Zhang A, Zhou A, Yuan J (2016). Association between human papillomavirus type 16 E6 and E7 variants with subsequent persistent infection and recurrence of cervical high-grade squamous intraepithelial lesion after conization. J Med Virol.

[16] Escobar-Escamilla N, González-Martínez BE, Araiza-Rodríguez A, Fragoso-Fonseca DE, Pedroza-Torres A (2019). Mutational landscape and intra-host diversity of human papillomavirus type 16 long control region and E6 variants in cervical samples. Arch Virol.

[17] Cornet I, Gheit T, Clifford GM, Combes J-D, Dalstein V (2013). Human papillomavirus type 16 E6 variants in France and risk of viral persistence. Infect Agent Cancer.

[18] Gauthier B, Cerigo H, Coutlée F, Franco EL, Brassard P (2018). Persistence of human papillomavirus 16, 18 and 52 variants in Inuit women from Northern Quebec, Canada. Int J Circumpolar Health.

[19] Aho J, Hankins C, Tremblay C, Forest P, Pourreaux K (2004). Genomic polymorphism of human papillomavirus type 52 predisposes toward persistent infection in sexually active women. J Infect Dis.

[20] Sun Z, Lu Z, Liu J, Wang G, Zhou W (2012). Genomic polymorphism of human papillomavirus type 52 in women from Northeast China. Int J Mol Sci.

[21] Ishizaki A, Matsushita K, Hoang HTT, Agdamag DM, Nguyen CH (2013). E6 and E7 variants of human papillomavirus-16 and -52 in Japan, the Philippines, and Vietnam. J Med Virol.

[22] Schlecht NF, Diaz A, Nucci-Sack A, Shyhalla K, Shankar V (2021). Incidence and types of human papillomavirus infections in adolescent girls and young women immunized with the human papillomavirus accine. JAMA Netw Open.

[23] Guo F, Hirth JM, Berenson AB (2015). Comparison of HPV prevalence between HPV-vaccinated and non-vaccinated young adult women (20-26 years). Hum Vaccin Immunother.

[24] Kumar S, Stecher G, Li M, Knyaz C, Tamura K (2018). MEGA X: Molecular Evolutionary Genetics Analysis across computing platforms. Mol Biol Evol.

[25] Burk RD, Harari A, Chen Z (2013). Human papillomavirus genome variants. Virology.

[26] Zhang Y, Cao M, Wang M, Ding X, Jing Y (2016). Genetic variability in E6, E7, and L1 genes of human papillomavirus genotype 52 from Southwest China. Gene.

[27] Bravo IG, Alonso A (2004). Mucosal human papillomaviruses encode four different E5 proteins whose chemistry and phylogeny correlate with malignant or benign growth. J Virol.

[28] Narechania A, Chen Z, DeSalle R, Burk RD (2005). Phylogenetic incongruence among oncogenic genital alpha human papillomaviruses. J Virol.

[29] Shah SD, Doorbar J, Goldstein RA (2010). Analysis of host-parasite incongruence in papillomavirus evolution using importance sampling. Mol Biol Evol.

[30] Gong Y, Sui L, Li Y (2022). Recombination in papillomavirus: controversy and possibility. Virus Res.

[31] Chen Z, Schiffman M, Herrero R, Desalle R, Anastos K (2011). Evolution and taxonomic classification of human papillomavirus 16 (HPV16)-related variant genomes: HPV31, HPV33, HPV35, HPV52, HPV58 and HPV67. PLoS One.

[32] Gagnon S, Hankins C, Money D, Pourreaux K (2007). Polymorphism of the L1 capsid gene and persistence of human papillomavirus type 52 infection in women at high risk or infected by HIV. J Acquir Immune Defic Syndr.

[33] Chen Z, Terai M, Fu L, Herrero R, DeSalle R (2005). Diversifying selection in human papillomavirus type 16 lineages based on complete genome analyses. J Virol.

[34] Chen Z, Schiffman M, Herrero R, DeSalle R, Anastos K (2013). Evolution and taxonomic classification of alphapapillomavirus 7 complete genomes: HPV18, HPV39, HPV45, HPV59, HPV68 and HPV70. PLoS One.

[35] Martínez MA, Jordan-Paiz A, Franco S, Nevot M (2019). Synonymous genome recoding: a tool to explore microbial biology and new therapeutic strategies. Nucleic Acids Res.

[36] Oyervides-Muñoz MA, Pérez-Maya AA, Sánchez-Domínguez CN, Berlanga-Garza A, Antonio-Macedo M (2020). Multiple HPV infections and viral load association in persistent cervical lesions in Mexican women. Viruses.

[37] De Brot L, Pellegrini B, Moretti ST, Carraro DM, Soares FA (2017). Infections with multiple high-risk HPV types are associated with high-grade and persistent low-grade intraepithelial lesions of the cervix. Cancer Cytopathol.

[38] Wang X, Wu S, Li Y (2021). Risks for cervical abnormalities in women with non-16/18 high-risk human papillomavirus infections in south Shanghai, China. J Med Virol.

[39] Hajia M, Sohrabi A (2018). Possible synergistic interactions among multiple HPV genotypes in women suffering from genital neoplasia. Asian Pac J Cancer Prev.

[40] Brant AC, Menezes AN, Felix SP, Almeida LM, Moreira MAM (2020). Preferential expression of a HPV genotype in invasive cervical carcinomas infected by multiple genotypes. Genomics.

[41] Zhou D, Dejnirattisai W, Supasa P, Liu C, Mentzer AJ (2021). Evidence of escape of SARS-CoV-2 variant B.1.351 from natural and vaccine-induced sera. Cell.

[42] Garcia-Beltran WF, Lam EC, St Denis K, Nitido AD, Garcia ZH (2021). Multiple SARS-CoV-2 variants escape neutralization by vaccine-induced humoral immunity. Cell.

